# Human Metapneumovirus Infection among Children, Bangladesh

**DOI:** 10.3201/eid1310.070337

**Published:** 2007-10

**Authors:** W. Abdullah Brooks, Dean Erdman, Pauline Terebuh, Alexander Klimov, Doli Goswami, Amina Tahia Sharmeen, Tasnim Azim, Stephen Luby, Carolyn Bridges, Robert Breiman

**Affiliations:** * ICDDR,B, Dhaka, Bangladesh; † Johns Hopkins University, Baltimore, Maryland, USA; ‡ Centers for Disease Control and Prevention, Atlanta, Georgia, USA; § Georgia Department of Human Resources, Atlanta, Georgia, USA; ¶ Centers for Disease Control and Prevention, Nairobi, Kenya

**Keywords:** Human metapneumovirus, pneumonia, children, paramyxoviridae, Dhaka, Bangladesh, dispatch

## Abstract

We confirmed circulation of human metapneumovirus (HMPV) among children with febrile and respiratory illness in an urban slum in Dhaka, Bangladesh, during active surveillance in 2001. HMPV was the most common single virus identified among febrile children and appears to contribute to the high rates of illness in this population.

Human metapneumovirus (HMPV) is the newest member of the family *Paramyxoviridae*, in the subfamily *Pneumovirinae*, shared with respiratory syncytial virus (RSV) (*1*). It appears to have 2 distinct genetic subgroups (*2**,**3*). HMPV was first described in a population of children in the Netherlands in 2001 (*1*) and has subsequently been linked with lower respiratory tract illness (LRTI) in children and adults (*2**,**4*). Although HMPV independently contributes to LRTI, some studies report more severe cases when HMPV is a coinfectant with RSV (*5**,**6*) or influenza (*7*); other studies have found no synergy (*3*).

## The Study

As previously reported (*8*), we undertook fever surveillance in Kamalapur, an urban community in Dhaka used by the International Center for Diarrheal Disease Research, Bangladesh (ICDDR,B) as a field site since 1998. The site has 7 geographic strata and 379 clusters. We randomly selected clusters within strata and enrolled all households within those clusters for surveillance, after obtaining informed written consent.

Field research assistants (FRAs) screened for fever across all ages among 5,000 households once weekly using standardized calendar questionnaires. FRAs referred children <13 years of age who reported fever for any duration, or anyone >13 years who reported fever for >3 days, to our onsite clinic where study physicians conducted standardized history and physical examinations. If an axillary temperature of >38°C was confirmed, physicians collected 3–5 mL of blood from children <5 years and persons >5 years, respectively, as well as convalescent blood samples 14 days later. Blood samples were allowed to clot and then centrifuged to obtain serum.

We retrospectively selected serum samples to test for respiratory viruses from patients <13 years of age who had cough for 1–3 days and fever of >38.5°C; we also selected paired serum samples negative for dengue by immunoglobulin M antibody capture (MAC)–ELISA. These samples were sent to the Centers for Disease Control and Prevention (CDC; Atlanta, Georgia, USA) for testing by hemagglutination inhibition for influenza and enzyme immunoassay for RSV; parainfluenza types 1, 2, and 3; adenovirus; and HMPV by using standard methods (*9**,**10*). A positive acute HMPV infection was defined as a >4-fold rise in titer between acute-phase and convalescent-phase samples.

Statistical analysis was performed by using StataSE Release 9.2 (StataCorp, College Station, TX, USA). We compared continuous variables between groups by using analysis of variance. For univariate analysis of categorical variables, we used 2 × 2 tables; for multivariate analysis, we used conditional logistic regression to determine strength of association between HMPV infection and potential explanatory covariates to obtain relative odds (RO) and 95% confidence intervals (CIs); p values were obtained by using the Fisher exact test. This study was approved by the research review and ethical review committees of ICDDR,B and the Institutional Review Board of CDC.

From December 6, 2000, through December 5, 2001, 889 persons came to our clinic with fever, and blood samples were collected from 888 (99.9%). Of the 889, 775 (84.9%) were self-referred; 114 (93.4%) of 122 were referred by FRAs during the same period. Of the 888 sampled patients, we selected serum samples from 128 children <13 years of age who had paired samples, documented fever >38.5C, cough for 1–4 days before first blood collection, and negative test results for dengue antibodies by MAC-ELISA. These samples were tested by hemagglutination inhibition against influenza virus A (H1N1 and H3N2) and influenza type B. Among these, 107 paired samples had sufficient remaining serum to be tested for other respiratory viruses, including HMPV.

Table 1 shows the distribution of all virus infections detected by serologic testing of 107 paired specimens. Of 60 infections detected among these children, 20 (33.3%) were caused by HMPV, the largest single group after influenza (although more than either influenza A or B alone). HMPV was detected in the dry premonsoon season from January through the end of June (Figure).

**Table 1:** Viruses detected in children <13 years of age by serology, Kamalampur, Bangladesh, December 2000–December 2001

Virus	No. infections	% (N = 60)	Cumulative %
Human metapneumovirus	20	33.3	33.3
Respiratory syncytial virus	3	5.0	38.3
Adenovirus	4	6.7	45.0
Parainfluenza virus 3	9	15.0	60.0
Influenza (H1N1)	8	13.3	73.3
Influenza (H3N2)	2	3.3	76.6
Influenza B	14	23.3	99.9

**Figure:**
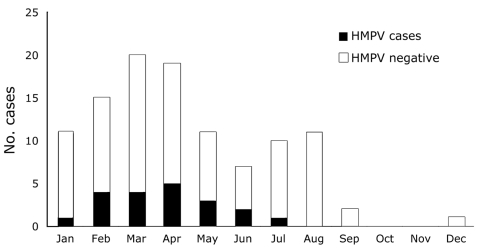
Human metapneumovirus (HMPV) infection in children <13 years of age, Kamalapur, Bangladesh, 2001.

We found no demographic differences in subgroup analysis by age group (<5 years and >5 years) or between children with acute HMPV infection and noninfected children (Table 2). Also, no differences were found in the reported history of fever duration or other complaints associated with febrile or respiratory illness in this population before treatment.

**Table 2:** Demographic and clinical characteristics of children <13 years of age presenting with fever, Kamalampur, Bangladesh, December 2000–December 2001*

Variables†	HMPV positive (N = 20)	HMPV negative (N = 87)	Relative odds (95% CI)	p value‡
Mean age, y§ (SD, 95% CI)	4.5 (2.6; 3.2–5.7)	4.5 (3.1; 3.9–5.2)	–	0.951
Mean age for 0–4 age range, y§ (SD, 95% CI)	2.9 (1.4; 2.1–3.8)	2.3 (1.2; 2.0–2.7)	–	0.113
Mean age for 5–12 age range, y§ (SD, 95% CI)	7.3 (1.9; 5.5–9.0)	7.7 (1.8; 7.1–8.4)	–	0.556
Children <5 y, no. (%)	13 (65.0)	52 (59.8)	1.25 (0.41–4.08)	0.801
Male gender, no. (%)	9 (45.0)	49 (56.3)	0.63 (0.21–1.89)	0.457
Duration of fever prior to clinic,§ d (SD, 95% CI)	3.1 (0.8; 2.7–3.5)	3.1 (1.7; 2.7–3.4)	–	0.983
Symptoms				
Headache, no. (%)	8 (40.0)	34 (39.1)	1.04 (0.33–3.10)	1.000
Body pain, no. (%)	4 (20.0)	25 (28.7)	0.62 (0.14–2.20)	0.580
Rhinorrhea, no. (%)	16 (80.0)	60 (69.0)	1.80 (0.51–8.06)	0.419
Difficulty breathing, no. (%)	3 (15.0)	4 (4.6)	3.66 (0.48–23.48)	0.119
Normal activity/behavior, no. (%)	15 (75.0)	64 (73.6)	0.73 (0.22–2.20)	0.620
Fever,§ °C (SD, 95% CI)	39.2 (0.6; 39.0–39.5)	39.1 (0.6; 39.0–39.2)	–	0.899
High fever (>39°C), no. (%)	10 (50.0)	49 (56.3)	0.78 (0.26–2.32)	0.627
Respiratory rate§ (SD, 95% CI)	42 (11; 36–47)	41 (9; 39–43)	–	0.808
Crepitations (rales) or wheezing, no. (%)	11 (55.0)	28 (32.1)	2.57 (0.85–7.86)	0.072
Altered mental status,¶ no. (%)	5 (25.0)	8 (9.2)	3.29 (0.73–13.19)	0.065
Altered mental status¶ if <5 y#, no. (%)	5 (38.5)	6 (11.5)	4.79 (0.90–23.86)	0.035
Pneumonia/LRTI diagnosis, no. (%)	8 (40.0%)	14 (16.1)	3.48 (1.02–11.24)	0.029

*HPMV, human metapneumovirus; CI, confidence interval; SD, standard deviation; LRTI, lower respiratory tract illness. †Categorical variables report N persons (percent of persons with characteristic in each group). Compared by 2 x 2 table, odds ratio with 95% CI. ‡2-tailed Fisher exact text. §Continuous variables. Means compared by using Student t test. ¶Irritability, lethargy. #N = 65 children <5 y.

Clinical findings (Table 2) showed no differences in mean fever or proportion of children with high fever (>39°C). However, compared with non-HMPV infection, acute HMPV infection was 3.5 times more likely (95% CI 1.02–11.24) to be associated with clinical pneumonia in all children and 4.8 times more likely (95% CI 0.90–23.86) to be associated with altered mental status (irritability/lethargy) in children <5 years old (Table 2). Only 1 child with acute HMPV infection had coinfection with another virus (influenza A).

## Conclusions

To our knowledge, ours is the first reported finding of HMPV in Bangladesh demonstrating substantial contribution of HMPV to febrile and lower respiratory tract illness in children <13 years of age. Our report substantiates that of a study from India (*11*). In a hospital study conducted in Bangladesh, a virus was isolated in only 33.3% of children with LRTI (*12*). Although HPMV was unknown at that time, and thus would not have been reported, the contribution of viruses to LRTI in children is often underreported by studies that have focused on bacterial infection identification or that did not include collection of paired serum samples to detect viral infections (*3*). HMPV has likely been a major factor in pneumonia and bronchiolitis in this population, as it has in others (*1**,**2**,**11*). In this study, HMPV was not only significantly associated with pneumonia, but with lethargy, an indicator of severe pneumonia in young children. Given the high rates of illness and death from pneumonia in this population (*8*), this association has important implications for disease control strategies. HMPV was also found in the dry pre-Monsoon season, when incidence of pneumonia peaks in this population. Similarly, parainfluenza peaks from March–April. In contrast, influenza occurs before and during the Monsoon season (March–August).

Our pilot study to assess the possible effects of HMPV on LRTI in children in Bangladesh had the following limitations: 1) healthy control patients were not included in the study; 2) the study was not originally designed to look for respiratory viruses; 3) fever was a main selection criterion and may have biased selection of more severe illnesses (a previous pneumonia study indicated that <33% of children in this environment with severe pneumonia have fever (*13*), perhaps substantially underestimating HMPV prevalence); 4) the observation period of 1 year may not represent the typical seasonal pattern; 5) case identification was based on serologic test results, and some children may have had a subclinical immune response or acute-phase samples may have been collected too late to observe a significant rise in titer, thus underestimating prevalence of disease; and 6) the study included insufficient cases to analyze viral interaction. To more clearly define the role of HMPV and other respiratory viruses in this population, and to improve disease control strategies, fever surveillance targeting a broader range of clinical syndromes over a sustained period is needed.
